# Photoaging Elevated the Genotoxicity of Polystyrene Microplastics to Marine Mussel *Mytilus trossulus* (Gould, 1850)

**DOI:** 10.3390/ijms25115740

**Published:** 2024-05-24

**Authors:** Victor Pavlovich Chelomin, Valentina Vladimirovna Slobodskova, Nadezhda Vladimirovna Dovzhenko, Andrey Alexandrovich Mazur, Sergey Petrovich Kukla

**Affiliations:** Il’ichev Pacific Oceanological Institute, Far Eastern Branch, Russian Academy of Sciences, 690041 Vladivostok, Russia

**Keywords:** polystyrene, UV irradiation, DNA damage, cytotoxicity, mussels

## Abstract

Micro-sized particles of synthetic polymers (microplastics) are found in all parts of marine ecosystems. This fact requires intensive study of the degree of danger of such particles to the life activity of hydrobionts and needs additional research. It is evident that hydrobionts in the marine environment are exposed to microplastics modified by biotic and abiotic degradation. To assess the toxic potential of aging microplastic, comparative studies were conducted on the response of cytochemical and genotoxic markers in hemocytes of the mussel *Mytilus trossulus* (Gould, 1850) after exposure to pristine and photodegraded (UV irradiation) polystyrene microparticles (µPS). The results of cytochemical tests showed that UV-irradiated µPS strongly reduced metabolism and destabilized lysosome membranes compared to pristine µPS. Using a Comet assay, it was shown that the nuclear DNA of mussel hemocytes showed high sensitivity to exposure to both types of plastics. However, the level of DNA damage was significantly higher in mussels exposed to aging µPS. It is suggested that the mechanism of increased toxicity of photo-oxidized µPS is based on free-radical reactions induced by the UV irradiation of polymers. The risks of toxic effects will be determined by the level of physicochemical degradation of the polymer, which can significantly affect the mechanisms of toxicity.

## 1. Introduction

The global production of synthetic polymers (plastic) continues to grow despite many concerns on the part of ecologists. End-of-life plastic products overflow into numerous landfills, are carried by the wind and various waterways, and eventually into the marine environment. Coastal currents carry litter into open waters where they become involved in giant oceanic gyres that form huge accumulations of countless plastic items [1,2,3].

Throughout the migration in the environment, plastic fragments are constantly subjected to physicochemical and biological influences. The specific environment into which the plastic is exposed determines the dominance and intensity of each individual factor or set of factors. In the process of these influences, polymer structures gradually lose their primary properties, become rigid, brittle, and disintegrate into small fragments of various sizes. Such particles ranging in size from a few millimeters to a few micrometers (less than 5 mm), as suggested by Thompson and colleagues [4], were named “microplastics”.

Large fragments of plastic products pose a real threat to large marine organisms (i.e., marine mammals, seabirds, and various species of turtles). At the same time, microplastics (MPs) are accessible to a very wide range of different taxa of different trophic levels from fish and invertebrates to relatively small planktonic organisms [5,6,7,8,9,10].

At present, the ability of MPs to passively or actively penetrate into the digestive system and tissue cells of various marine organisms has become a subject of special attention of ecotoxicologists. There is a risk not only of introducing synthetic polymers into various food chains [11,12,13], but also of initiating toxic threats. With increasing experimental data, it is becoming increasingly clear that microparticles of different types of synthetic polymers cause multiple negative changes in physiological and biochemical processes in various representatives of marine organisms [14,15,16,17,18,19]. The reasons for such diverse display of negative properties of MPs are not fully understood, although the importance of research in this direction is undoubted, given the presence of plastic fragments in all ecosystems.

From the analysis of most of the laboratory experiments, it follows that the negative effects of MPs are shown in the example of pristine plastic samples, which is significantly different from the situation occurring in real natural conditions. From the analysis of most of the laboratory experiments, it follows that the negative effects of MPs are shown on the example of pristine plastic samples, which is significantly different from the situation occurring in real natural conditions. In reality, organisms interact with plastic fragments that are in the process of abiotic (physicochemical) and biotic degradation, the detailed mechanisms of which are still poorly understood. It is only known that in the marine environment, the fragmentation process of synthetic polymers is very complex and occurs as a result of physical and chemical changes initiated by UV radiation (sunlight) followed by the oxidative activity of oxygen (atmospheric and dissolved), hydrolytic action of water, and catalytic influence of soluble forms of metals with variable valence [20,21]. In laboratory conditions, it is difficult to recreate in full the entire complex of natural factors involved in the degradation of synthetic polymers; therefore, in experimental works, methodological approaches involving UV irradiation are actively introduced, which best simulate the main natural conditions that lead to photo-oxidative and hydrolytic reactions in polymers [22]. This approach, called the “artificial aging” of plastics, is now widely used to accelerate degradation processes and to study the physicochemical properties of degrading plastics [23,24,25,26,27].

Because of UV irradiation, additional O_2_-containing functional groups appear in the aging polymer structure. In addition, polymer chains shorten, crystallinity decreases, and hydrophilic properties increase, which significantly affects the mechanical, physical, and chemical properties of the polymer [23,26,27,28,29,30]. Therefore, from an ecotoxicological point of view, studies of possible negative effects of hydrobionts’ interaction with aging MPs of different degradation degrees are of particular relevance [31]. The research involving artificial aging MPs, although few in number, shows that such particles and their extracts have serious effects on various cellular processes in living organisms [32,33,34,35,36,37]. In addition, evidence has been presented that degraded fragments are more toxic than pristine plastic samples [38,39,40].

Despite the fact that aging MPs are widely represented in the marine environment, their role in the development of toxic processes in hydrobionts is poorly understood and needs further research. In the interests of developing this direction, we conducted comparative experiments of the effect of pristine and artificial aging (using UV irradiation) polystyrene microfragments (µPS) on the viability and stability of the genome of hemocytes of the mussel *Mytilus trossulus* (Gould, 1850). Among a wide variety of marine organisms, filter-feeding mollusks, in particular bivalves of the family Mytilidae, are of particular interest from the point of view of studying the mechanisms underlying the toxic effects of MPs. In the process of feeding, mollusks, while filtering large volumes of water, inevitably extract, concentrate, and retain abiotic particles of anthropogenic source, including plastic particles, along with food particles [41,42]. We consider that studies of genotoxic characteristics provide an opportunity to predict changes at the subcellular level and make a significant contribution to the establishment of causal relationships in the interaction of an organism with any toxicant, including MPs.

## 2. Results

**Figure 1:**
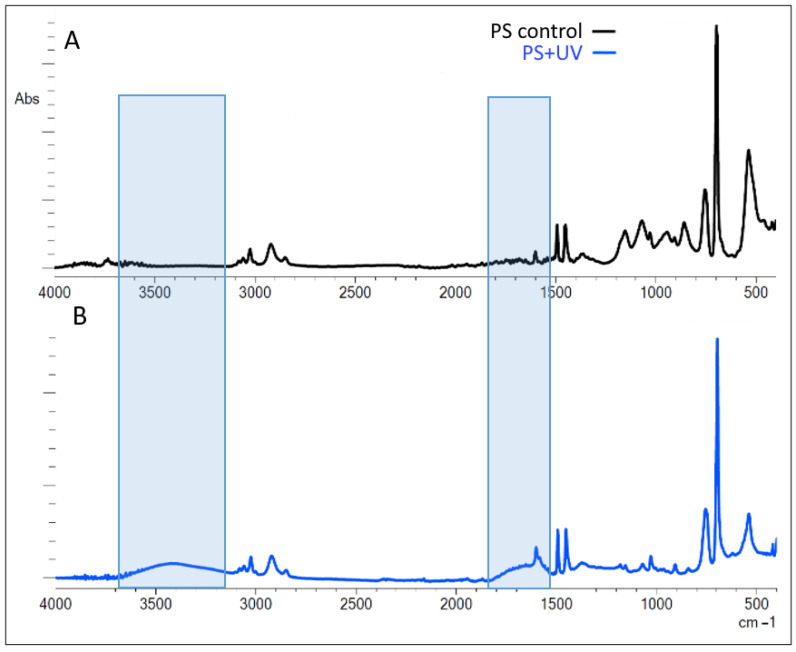
FTIR spectra of pristine (**A**) and artificial aging (**B**) polystyrene microgranules for 120 h with UV irradiation. Blue rectangles indicate the areas of spectra with the largest changes.

FTIR spectra of samples of pristine and photodegraded polystyrene microgranules (µPS) are shown in Figure 1.

According to FTIR data, the pristine µPS (Figure 1A) had spectral bands characteristic of this polymer: 1600 cm^−1^, 1492 cm^−1^, and 1452 cm^−1^, indicate to vibrations of aromatic C=C bonds, as well as 3026 cm^−1^ (vibrations of valence aromatic C–H bonds), which indicates the presence of benzene rings. In addition, these samples present 698 cm^−1^ and 756 cm^−1^ peaks arising from out-of-plane strain vibrations of C–H bonds, which is characteristic of the presence of substituents in the benzene ring.

After continuous UV irradiation for 120 h, significant changes are observed in the µPS spectrum (Figure 1B) due to the appearance of a new peak in the range of 3100–3600 cm^−1^ indicate to O–H bond vibrations (hydroxyl group) and an increase in the area of peaks in the range of 1600–1750 cm^−1^ consistent with C=O bonds (carbonyl group). These spectral lines in FTIR spectra indicate the development of oxidation processes in polymer chains of µPS induced by UV irradiation.

Indices reflecting the degree of oxidative degradation of polystyrene chains under UV irradiation under our experimental conditions are presented in Table 1. Based on these indices, it can be seen that after UV irradiation, the content of carbonyls (C=O bonds) and hydroxyls (O–H bonds) in the composition of PS chains increased almost three-fold and ten-fold, respectively.

**Figure 2:**
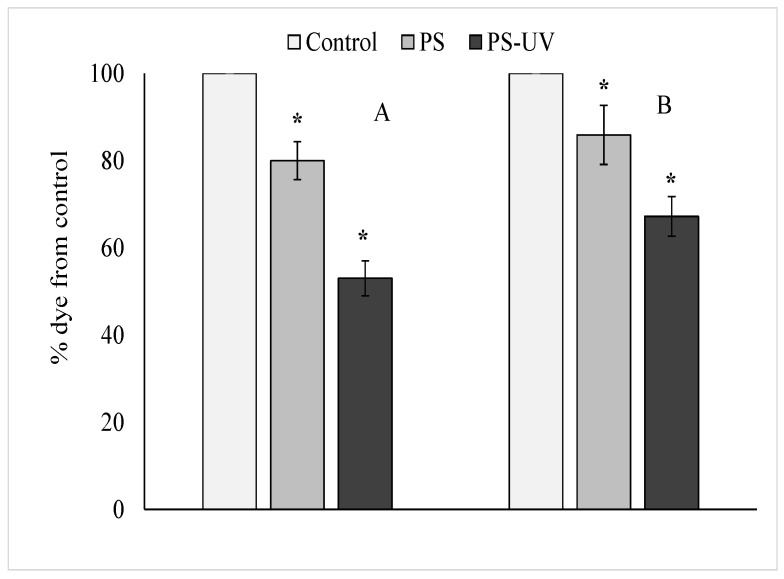
Determination of viability of mussel *Mytilus trossulus* hemocytes in the control and experimental groups using a resazurin test (**A**) and neutral red dye (**B**) (mean ± standard deviation, *n* = 9), *—difference from control is significant at *p* < 0.05.

The results of analyzing the viability of hemocytes of control and experimental groups of mussels are shown in Figure 2. The resazurin assay showed that, compared to hemocytes of control mollusks, hemocytes of experimental mussels exposed to pristine and UV-irradiated µPS significantly decreased the ability to recover resazurin into resorufin (Figure 2A). These results indicate a decrease in the metabolic activity of hemocytes from both experimental groups of mollusks. Furthermore, it is evident from the results of these experiments that UV-irradiated µPS had a greater effect compared to pristine particles.

**Figure 3:**
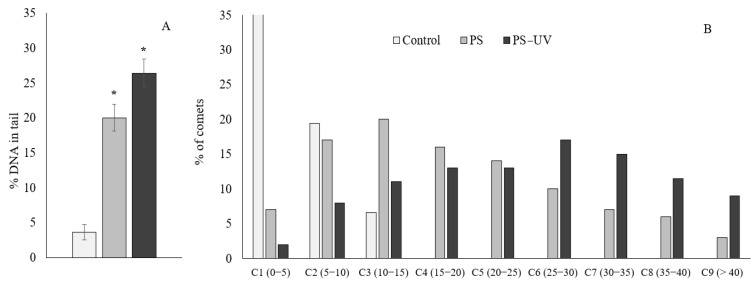
The DNA damage level of hemocytes of mussel *Mytilus trossulus* hemocytes in the control and experimental groups. Value of % DNA in the comet tail (**A**) and distribution of comets according to the degree of fragmentation with an interval of 5% (**B**) (mean ± standard deviation, *n* = 20), *—difference from control is significant at *p* < 0.05.

A similar trend was observed when the cytochemical test using neutral red dye was used (Figure 2B). The results show that hemocytes from the experimental groups of mussels have a significantly decreased ability to retain the dye, indicating destabilization of lysosome membranes induced by uptake of both types of µPS. As in the case of the resazurin test, UV-irradiated PS microgranules had a greater destabilizing effect on lysosome membranes compared to pristine µPS. To assess the level of destructive processes in the genome of hemocytes of control and experimental mollusks after exposure to both types of microplastic, we applied the DNA comet assay, which allows us to evaluate this parameter in individual cells. Figure 3A shows one of the quantitative parameters of the obtained comets (average values of % of DNA migrating in the electric field to the “tail” of the comet), which characterizes the level of damage of the DNA molecule.

According to the data of comet analysis, a low level (<4.5%) of nuclear DNA fragmentation was observed in hemocytes of mussels kept in aerated seawater without the addition of polystyrene microgranules. From the presented data, it can be seen that in hemocytes of the experimental groups of mollusks in whose aquariums pristine µPS was present, the average level of damaged DNA increased significantly (>4-fold) compared to control mollusks. In addition, it should be emphasized that in experimental mussels in the presence of UV-irradiated µPS, this index was even more pronounced compared to the hemocytes of mollusks exposed to pristine polystyrene microgranules (Figure 3A).

For a more detailed analysis of the nature of the comet genome destruction, the comets that are formed from the DNA of hemocytes of the control and experimental mussels were grouped according to the degree of fragmentation (with a 5% interval), and are presented in Figure 3B. In the control mussel groups, the proportion of hemocytes with an upper limit of destruction of nuclear DNA (10–15%) is about 5–6%. Whereas in experimental mussels, after exposure to both types of µPS (pristine and UV-irradiated), hemocytes with a high degree of DNA damage are present, reaching 35–40% (C8) and more (C9).

## 3. Discussion

With the accumulation of experimental data, it is increasingly evident that polymers aging in artificial or natural environments are more bioactive. According to experimental data, µPS aged under natural or artificial conditions (UV irradiation) had a greater effect on physiological [19,33,34,43] and biochemical [15,18,19,44,45] processes compared to pristine µPS. The results of our studies on the mussel *Mytilus trossulus* (Gould, 1850) confirm this trend. The µPS aging induced by UV irradiation leads to an increase in their biological activity, which is manifested in the enhancement of their cyto- and genotoxic properties compared to pristine particles.

To evaluate the response of mussels to both µPS, we applied sensitive cytochemical tests related to general stress, which are widely used as bioindicators of the current state of mollusk health [2,46,47]. As shown by the resazurin test in the hemocytes of both experimental groups of mussels, the intensity of resazurin recovery decreased, indicating the suppression of general metabolism in these cells [48]. Moreover, this effect was more significant in mussels after exposure to aging µPS. The hemocytes of this group also showed the lowest level of neutral red dye retention due to a significant decrease in the stability of lysosome membranes (Figure 2B).

Analysis of the influence of µPS on DNA integrity showed similar results. It should be noted that in hemocytes of mussels maintained in water without the addition of polystyrene particles, a relatively low level of nuclear DNA fragmentation (<4.5%) was observed, which is formed during the functioning of life-supporting systems, mainly due to the accumulation of single- and double-stranded breaks and the accumulation of alkali-labile sites [49]. According to comet assay data in experimental mollusks, the level of DNA damage increased significantly after exposure to plastic, with UV-irradiated µPS inducing more significant DNA damage to hemocytes than in pristine. This was particularly pronounced when analyzing the distribution of comets by DNA damage level (Figure 3B). In experimental mussels, after exposure to both types of µPS, there are hemocytes with a high degree of DNA damage, reaching 35–40% (C8) and more (C9). However, in mussels exposed to pristine µPS, the main part of hemocytes is represented by C2–C4 classes with DNA damage levels ranging from 5 to 25%, whereas in mussels exposed to aging µPS, hemocytes with a level of genome destruction ranging from 20 to 40% dominate.

Based on these results, it can be argued that a relatively high degree of DNA damage initiated by µPS, especially after photo-oxidation, increases the risk of further spread of destructive processes in cells. The above experimental results suggest that the mussel hemocyte is a sensitive target for µPS exposure. Moreover, considering that hemocytes are a key component of the detoxification and immune systems [50], the DNA damage of these cells may be a trigger in the development of serious biochemical and physiological damage resulting in toxic effects on the whole organism.

Although it is beyond the scope of the current work to investigate this issue, this argument is deserving of mention because it is predictive of the development of remote harmful effects at the ecological level. To better understand the mechanisms and consequences of the effects of aging µPS on biological systems, it is necessary to evaluate the changes occurring in the polymer structure under UV irradiation.

In the present study, µPS were exposed to a continuous UV source (290–360 nm, 25 W/m^2^) for 120 h. This exposure time is equivalent to two weeks of solar irradiation under natural conditions, with the most approximate calculations [43,51]. In addition, the UV irradiation energy of our source is sufficient to dissociate C–C and C–H bonds [52].

To evaluate UV-induced changes in µPS, we used FTIR, which is widely used today as the main tool to detect and evaluate chemical changes in the structure of artificial polymers [53,54,55]. It is well known that the aging process of polymers initiated by UV exposure starts at the surface; therefore, chemical changes recorded with FTIR characterize mainly the degradation of surface polymer chains.

According to FTIR spectrometry data (Figure 1), UV exposure of µPS leads to the appearance or enhancement of spectral bands, indicating the formation of mainly carbonyl and hydroxyl functional groups in the structure of polymer chains. Thus, the enhancement of a broad spectral line in the region 1635–1765 cm^−1^, representing the known carbonyl domain, reflects the formation of several products including ketone, carbonic, and aldehyde functional groups [53,55]. The appearance of a new peak in the FTIR spectra in the range of 3420–2550 cm^−1^ corresponds to vibrations of the hydroxyl bond (O–H) in different functional groups [56].

The formation of these oxygen-containing functional groups indicates the development of oxidative degradation processes of polymer chains, which are characteristic of both artificial and natural marine conditions aging in PS fragments [21,24,45,57]. It has also been found that photo-oxidation of polystyrene leads to polymer chain breakage reactions and the formation of various chemical products that leach into the environment [26,30,58].

Such changes in chemical structure have a significant impact on the physical and mechanical characteristics of various polymers, including PS [53,59]. A number of experimental works have shown that UV irradiation of polymers leads to an increase in crystallinity, the number of surface pores and microcracks, changes in negative charge and hydrophilicity, as well as increased fragmentation and decreased particle size [18,26,44,52,53].

Physicochemical changes induced by UV irradiation in laboratory conditions and in the natural environment led to significant changes in the sorption characteristics of the polymer towards inorganic and organic substances [29,39,44,60]. As shown by Liu and colleagues [39], in pristine PS fragments, hydrophobic and π-π interactions are involved in the sorption of pharmacological drugs, whereas in artificially aged PS, electrostatic interactions and hydrogen bonds are dominant.

According to the shown complex of changes induced in the process of polymer aging, there is reason to assume that oxidative degradation of the polymer is the reason for the enhancement of its biological activity. It should be noted that the mechanisms of oxidative degradation of polymers are based on free-radical reactions [26,56,59,61] which can induce the formation of reactive oxygen species and make a certain contribution to the enhancement of pro-oxidant processes in biological systems. Although this assumption is hypothetical, this mechanism explains the facts of activation of oxidative stress processes reported in the literature [18,38,45,62] and the enhancement of cyto- and genotoxic properties of UV-irradiated µPS revealed in our study.

## 4. Materials and Methods

### 4.1. Description of the Experiment

Adults of *M. trossulus* (4.5–5 cm in shell height) were sampled in one mollusk communities of one generation in Alekseev Bay (Sea of Japan) by SCUBA divers. All experiments were carried out at a marine experimental station “Popov Island” of the V.I. Il’ichev Pacific Oceanological Institute. All procedures in the present work, as well as the mollusk disposal methods, were approved by the Commission on Bioethics at the V.I. Il’ichev Pacific Oceanological Institute, Far Eastern Branch of Russian Academy of Science (protocol №16 and date of approval 15 April 2021), Vladivostok, Russia. After acclimation, all mussels were divided into three groups: one group without treatment (control group), a second group with pristine µPS, and a third group with aging (UV irradiation) µPS for up to 5 days. In each group, a control and two experimental and three parallel tanks with a volume of 20 L were used. There were 10 mussels in each of them so that the stocking density was 1 mollusk per 2 l of seawater. Intensive air blowing was used to prevent microplastic from settling to the bottom and to maintain a stable oxygen concentration in the water. During the entire experiment, including adaptation, environmental conditions were maintained at constant (T 17–18 °C, pH 8.2 ± 0.1; salinity 32.52 ± 0.21 psu; O_2_ 7.5 ± 0.3 mg/l and photoperiod 16 h light: 8 h dark). The animals were not fed additionally. Mussel hemocytes were used for all analyses. The number of hemocytes in the selected hemolymph of *M. trossulus* was counted under a microscope AxioImager A1 (Zeiss, Oberkochen, Germany) using a Goryaev chamber with 5 repetitions. The cell concentration in all studied cells was 4 ± 0.5 × 10^6^ mL. During the experimental period, the seawater was changed every 24 h.

A working solution µPS with a concentration of 10^6^ microspheres/l (diameter 0.9 µm) was prepared using a commercial solution with a concentration of 5% *w*/*v* (BaseLine ChromTech Research Centre, Tianjin, China). To determine the working concentrations of microspheres in the solutions used, microsphere counting was carried out in a Goryaev chamber. The counting was carried out in triplicate for each tank.

UV irradiation of plastic particles was carried out in a Petri dish using a lamp Supratec HTC 400-241 (Osram, Munich, Germany) 460 W power for 120 h. The distance from the lamp to the surface of plastic particles was 3 cm.

### 4.2. Determination of Cytotoxicity

Cytotoxicity of the compounds tested was determined by resazurin cytotoxicity assay and neutral red assay (NR).

The NR assay is based on the uptake and accumulation of NR in the lysosomes of living cells. In damaged cells, the rate of NR accumulation changes, and dead cells are unable to retain the dye [63]. In total, 300 μl of hemolymph was collected from 3 mussels, in total there were 3 replicates for each study: control/µPS/µPS-UV, 9 individuals for each experimental group. Next, 60 μl of NR solution (50 μg/mL) was added to 300 μl of hemolymph and incubated for 1 h at a temperature of 37 °C on a thermoshaker TS-100C (Biosan, Riga, Latvia). Then, the cells were washed from the remaining dye twice with a 300 μL PBS solution (pH 7.4). Finally, 300 μl acetic acid ethanol solution (1% acetic acid, 50% ethanol) was added and incubated at 20 °C for 15 min. The absorbance was measured at 540 nm using a spectrophotometer UV-2550 (Shimadzu, Kyoto, Japan). Cell viability was expressed as a percentage of cell survival compared to control [64].

The Resazurin cytotoxicity assay is based on the ability of living cells to convert a blue non-fluorescent dye, resazurin, into a pink fluorescent dye, resorufin, which makes it possible to determine the cytotoxicity of cells colorimetrically or fluorimetrically. Viable cells continuously convert resazurin to resorufin, increasing the overall fluorescence and color of the environment in which they are found. In our study, we applied the protocol described by [65]. Hemolymph was collected in the same way as for determining cytotoxicity using the NR method—300 μl was taken from 3 mussels, a total of 3 replicates for each study: control/µPS/µPS-UV, 9 individuals for each experimental group. Next, 30 μl of the 10X solution Resazurin in PBS (pH 7.4) was added to 300 μl of hemolymph and incubated for 1 h at a temperature of 37 °C on a thermoshaker TS-100C (Biosan, Riga, Latvia). For colorimetric analysis, absorbance was measured at 570 nm and at 600 nm using a spectrophotometer UV-2550 (Shimadzu, Kyoto, Japan).

### 4.3. Comet Assay

To determine the genotoxicity of polystyrene microparticles, an alkaline version of the comet assay was used [16]. In total, 50 μl of hemolymph was added to 100 μl of 1% low-melting agarose (MP Biomedicals, Eschwege, Germany) and applied to a glass slide previously coated with 1% agarose solution. After the gel hardened in the cold after 3 min, the slides were moved into a cooled lysis solution (2.5 M NaCl; 0.1 M EDTA-Na, 1% Triton X—100; 10% DMSO; 0.02 M Tris, pH 10) for 1 h in a dark, cool place. Next, the slides were removed, carefully washed with cooled distilled water, and transferred to electrophoresis buffer (300 mM NaOH, 1 mM EDTA-Na_2_) for 40 min. After 40 min, electrophoresis was carried out (in a dark, cool place) at a voltage of 2 V/cm for 20 min. Then, neutralization was immediately carried out (0.4 M Tris-HCl, pH 7.4) and the slides were washed with cold distilled water. After drying in air, the slides were stained with the fluorescent dye SYBR Green I, after which DNA comets were visualized and recorded using a scanning fluorescence microscope AxioImager A1 (Zeiss, Oberkochen, Germany) equipped with an AxioCam MRc digital camera. A computer program was used to process digital images CASP 1.2.2. program (CASPlab, Wroclaw, Poland, https://casplab.com, accessed on 20 May 2024).

The percentage of DNA damage (% DNA in tail) was determined for each comet. In the control and experimental groups, comets were counted for each mollusk in two parallels: 1 mollusk = 2 glass slides (*n* = 20) containing at least 50 comets.

Also, for greater clarity, the resulting DNA comets were divided into 9 classes with a damage interval of 5%: <5% DNA in tail (Class 1); 5–10% DNA in tail (Class 2); 10–15% DNA in tail (Class 3); 15–20% DNA in tail (Class 4); 20–25% DNA in tail (Class 5); 25–30% DNA in tail (Class 6); 30–35% DNA in tail (Class 7); 35–40% DNA in tail (Class); and comets with damage levels > than 40% were classified as Class 9.

### 4.4. Fourier-Transform Infrared (FTIR) Spectroscopy

FTIR spectra were acquired using an IRAffinity-1S (Shimadzu, Kyoto, Japan) equipped with an attachment-frustrated total internal reflection (wavenumber range of 4000–400 cm^−1^, 32 scans per spectrum, spectral resolution of 4 cm^−1^). The background was measured with the same settings against air. The obtained spectra were processed using LabSolutions IR 2.27 software (Shimadzu, Kyoto, Japan). The content of functional groups in all µPS samples was calculated using the following indices: carbonyl index (CI) [55], and hydroxyl index (HI) [56].

### 4.5. Statistical Analysis

The experiment results were processed in the MS Excel and Statistica 10 software packages (StatSoft, Tulsa, OK, USA). For the data, nonparametric Kruskal–Wallis ANOVA followed by pairwise Mann–Whitney tests were performed. A difference at *p* < 0.05 was considered to be statistically significant.

## 5. Conclusions

Plastic debris, including MPs, are at different stages of decomposition depending on the time of stay in the environment and intensity of exposure to physicochemical factors. Thus, to understand the risk of plastic pollution to marine organisms, it is necessary to know their response to exposure to modified “artificial aging” plastic. In the present study, the toxicity of pristine and UV-irradiated µPS on the bivalve mollusk *Mitylus trossulus* (Gould, 1850) was evaluated. The results of the study show that at penetration of MPs of artificial polymers into biological systems, the risks of negative consequences depend not only on their concentration, but also, to a certain extent, are conditioned by the level of physicochemical degradation of the polymer, which can significantly affect its toxicity mechanisms. The data obtained show the need for further investigation of the toxic effect of aging plastic particles on marine organisms.

## Figures and Tables

**Table 1:** Indexes of the content of functional groups in µPS samples.

Index	Formula	PS Control	PS UV Irradiation
CI	(1635–1650)/1452	0.39 ± 0.07	1.01 ± 0.12 *
HI	(3420–3550)/1452	0.03 ± 0.01	0.39 ± 0.04 *

*—difference from control is significant at *p* < 0.05.

## Data Availability

Data is contained within the article.
